# MRI texture features as biomarkers to predict MGMT methylation status in glioblastomas

**DOI:** 10.1118/1.4948668

**Published:** 2016-05-13

**Authors:** Panagiotis Korfiatis, Timothy L. Kline, Lucie Coufalova, Daniel H. Lachance, Ian F. Parney, Rickey E. Carter, Jan C. Buckner, Bradley J. Erickson

**Affiliations:** Department of Radiology, Mayo Clinic, 200 1st Street SW, Rochester, Minnesota 55905; Department of Radiology, Mayo Clinic, 200 1st Street SW, Rochester, Minnesota 55905; Department of Neurosurgery of First Faculty of Medicine, Charles University in Prague, Military University Hospital, Prague 128 21, Czech Republic; and International Clinical Research Center, St. Anne’s University Hospital Brno, Brno 656 91, Czech Republic; Department of Neurology, Mayo Clinic, 200 1st Street SW, Rochester, Minnesota 55905; Department of Neurologic Surgery, Mayo Clinic, 200 1st Street SW, Rochester, Minnesota 55905; Department of Health Sciences Research, Mayo Clinic, 200 1st Street SW, Rochester, Minnesota 55905; Department of Medical Oncology, Mayo Clinic, 200 1st Street SW, Rochester, Minnesota 55905; Department of Radiology, Mayo Clinic, 200 1st Street SW, Rochester, Minnesota 55905

**Keywords:** MRI, glioblastoma multiforme, MGMT, imaging biomarkers, support vector machines, random forest

## Abstract

**Purpose:**

Imaging biomarker research focuses on discovering relationships between radiological features and histological findings. In glioblastoma patients, methylation of the O^6^-methylguanine methyltransferase (MGMT) gene promoter is positively correlated with an increased effectiveness of current standard of care. In this paper, the authors investigate texture features as potential imaging biomarkers for capturing the MGMT methylation status of glioblastoma multiforme (GBM) tumors when combined with supervised classification schemes.

**Methods:**

A retrospective study of 155 GBM patients with known MGMT methylation status was conducted. Co-occurrence and run length texture features were calculated, and both support vector machines (SVMs) and random forest classifiers were used to predict MGMT methylation status.

**Results:**

The best classification system (an SVM-based classifier) had a maximum area under the receiver-operating characteristic (ROC) curve of 0.85 (95% CI: 0.78–0.91) using four texture features (correlation, energy, entropy, and local intensity) originating from the *T*2-weighted images, yielding at the optimal threshold of the ROC curve, a sensitivity of 0.803 and a specificity of 0.813.

**Conclusions:**

Results show that supervised machine learning of MRI texture features can predict MGMT methylation status in preoperative GBM tumors, thus providing a new noninvasive imaging biomarker.

## 1. INTRODUCTION

Glioblastoma multiforme (GBM) is the most common primary brain tumor accounting for 45% of all malignant primary central nervous system tumors with a median survival of around 14 months.^1^ GBMs are usually treated with surgical resection followed by radiation therapy and temozolomide chemotherapy, improving median survival by 3 months versus radiotherapy alone.^2^ MRI is most commonly used to assess response due to its superior contrast compared with other imaging modalities.^3^

Methylguanine methyltransferase (MGMT) is a key gene that encodes for a protein that repairs DNA. When it is methylated, the gene is inactivated, which suppresses DNA repair activity, including DNA in the tumor that is actively dividing. High tumor MGMT expression in patient samples is associated with TMZ resistance since tumor cells lacking MGMT activity are significantly more sensitive to the cytotoxic effects of TMZ. Thus, GBMs with MGMT promoter methylation can be expected to respond better to an alkylating agent like temozolomide.^4^ In addition, MGMT methylation may be considered as a predictive biomarker for a patient’s desirable response to radiation therapy. Several reports in the literature indicate that MGMT promoter methylation is associated with longer survival.^5^ However, while determination of MGMT methylation status has been standard of care for some time, an accurate result is not always obtained due to the requirement of large tissue specimens. Furthermore, there are a limited number of laboratories that are able to perform these tests.

An emerging hypothesis is that genetic and/or molecular alteration within GBM manifests as specific, macroscopic, observable changes in MRI anatomical imaging.^6^ Radiogenomics is an active area of research investigating the relationship between radiological and genomic features. Visual findings as well as texture features, originating from functional or anatomical MRI imaging, have been investigated as imaging biomarkers to predict MGMT status.^7–12^ Conversely, MGMT biomarker imaging along with other imaging features might facilitate optimal tissue sampling at surgery.

Moon *et al.*^10^ found that ill-defined tumor borders, lower attenuation coefficients in computed tomography scans, lower fractional anisotropy (FA), and increased apparent diffusion coefficient (ADC) values are associated with MGMT promoter methylation in a mixed group of WHO grade III and IV patients. Another study showed that ring enhancement correlates with unmethylated MGMT status.^8^ However, in a similar study by Gupta *et al.*,^12^ no correlation between MGMT and either ill-defined borders or perfusion imaging-based biomarkers was found. In a study of 43 patients by Ahn *et al.*,^11^ biomarkers based on ADC and FA parametric maps were found to be poor predictors of MGMT methylation, while capillary permeability (i.e., *Ktrans*) achieved an area under the receiver-operating characteristic (ROC) curve (AUC) of 0.756. It is unclear whether these differences are due to small sample sizes and spurious findings or whether different imaging properties are actually being measured, indicating a need for calculation standardization.

Up to now, only one study has exploited the use of texture features (i.e., variations of intensity that form certain repeated patterns) extracted from anatomical MRI images for their potential use as imaging biomarkers.^8^ Furthermore, the majority of studies use imaging biomarkers without evaluating combinations of them. Finally, the number of subjects is often small (<60), which can produce spurious results when machine learning methods are applied, particularly since the largest study (*N* = 77) showed no predictive ability.^12^

In this study, we utilize a dataset of preoperative MRI examinations in a larger number (*N* = 155) of GBM patients in order to evaluate the use of texture features as potential imaging biomarkers for predicting the MGMT methylation status of GBM tumors. The area under the ROC curve (*A_z_*) is used as the evaluation metric by which we evaluate the proposed classification schemes.

## 2. MATERIALS AND METHODS

### 2.A. Dataset

This study was reviewed and approved as minimal risk by our Institution’s Internal Review Board. Patients with newly diagnosed GBM (astrocytoma grade IV, WHO classification) treated at Mayo Clinic between January 1, 2007, and December 31, 2015, were identified. The inclusion criteria were age ≥18 yr and preoperative MR scans that included *T*2- and *T*1-weighted postcontrast images performed at Mayo Clinic with known MGMT methylation status. All the images were anonymized utilizing CTP (http://mircwiki.rsna.org/index.php?title=CTP-The_RSNA_Clinical_Trial_Processor), and the image processing pipelines were managed with MIRMAID.^13^

One hundred fifty-five presurgery MRI examinations were utilized in this study (66 methylated and 89 unmethylated tumors). MRI imaging was performed on 1.5 T or 3 T scanners and included *T*2-weighted fast spin-echo (TR, 4000–4800 ms; TE, 96–107 ms; slice thickness, 3 mm), axial *T*1-weighted images (TR, 20 ms; TE, 6 ms; slice thickness, 3 mm), with a FOV of 24 cm and a matrix size of 256 × 256, and matching *T*1-weighted postcontrast images. In all the exams, the contrast agent was power injected at 5 ml/s followed by a 20 cm^3^ saline chaser at the same flow rate. The contrast agent was gadolinium at 0.1 mmol/kg.

**FIG. 1.**
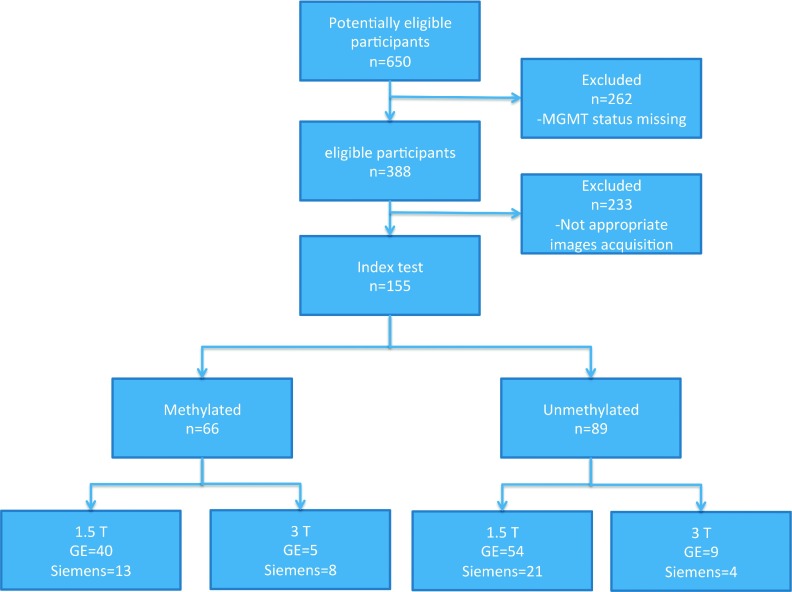
Diagram capturing dataset formation for this study.

From the 155 presurgery MRI examinations utilized in this study, 66 patients had methylated and 89 patients had unmethylated tumors. For the methylated group, 53 scans were performed on a 1.5 T scanner (40 GE and 13 Siemens), while 13 were performed on a 3 T scanner (5 GE and 8 Siemens). For the unmethylated group, 76 scans were performed on a 1.5 T scanner (54 GE and 21 Siemens), while 13 were performed on a 3 T scanner (9 GE and 4 Siemens). Each of the patients participating in this study had only one tumor (Fig. 1).

### 2.B. Image analysis

#### 2.B.1. Visual measurements

One neurosurgery resident, blinded to patients’ molecular data, reviewed the MR images and assessed the following tumor characteristics: enhancing tumor margin (well or poorly defined); enhancement pattern (ring, nodular, or mixed enhancement); presence of edema, cystic regions, necrosis, and nonenhancing tumor; and heterogeneity of the signal intensity on the *T*2-weighted images.^11,12^

Necrosis was defined as a region within the tumor enhancing area with little discernable contrast enhancement. Cystic regions were defined as areas isointense to CSF on *T*1- and *T*2-weighted images with homogeneous appearance with very thin enhancing rim on *T*1 postcontrast images surrounding at least 75% of the cystic region. Nonenhancing tumor was defined as an area of *T*2-weighted intermediate intensity (less than the intensity of cerebrospinal fluid or vasogenic edema, with corresponding *T*1 hypointensity) and at least 25% of the size of the enhancing part.

#### 2.B.2. Tumor segmentation

Enhancing tumor volumes were segmented on presurgical postcontrast *T*1-weighted images utilizing a semiautomatic technique. This technique used an automated algorithm to provide an initial segmentation of the enhancing part of the tumor followed by manual corrections. A neuroradiologist (using ITKsnap^14^) reviewed the results and corrected any oversegmentation or undersegmentation errors.

The algorithm that was implemented for the tumor segmentation was a supervised classification scheme utilizing random forest classifier (RFC) and co-occurrence texture features to differentiate between tumor enhancing parts and the surrounding tissue classes. The co-occurrence texture features used were correlation, energy, and entropy based on the co-occurrence matrix as well as the intensity extracted from 7 × 7 ROI. The system was developed utilizing 30 postcontrast *T*1-weighted images. Prior to feature extraction, intensity standardization was performed. The trained classifier was saved and was applied on the subjects participating in this study. For each subject, a sliding ROI was used to classify each area of the *T*1 scan as either tumor (enhancing region) or surrounding tissue. Subsequently, a morphological operation was performed to remove spurious regions. Both cystic regions and regions with significant nonenhancing macroscopic necrosis were excluded.

**FIG. 2.**
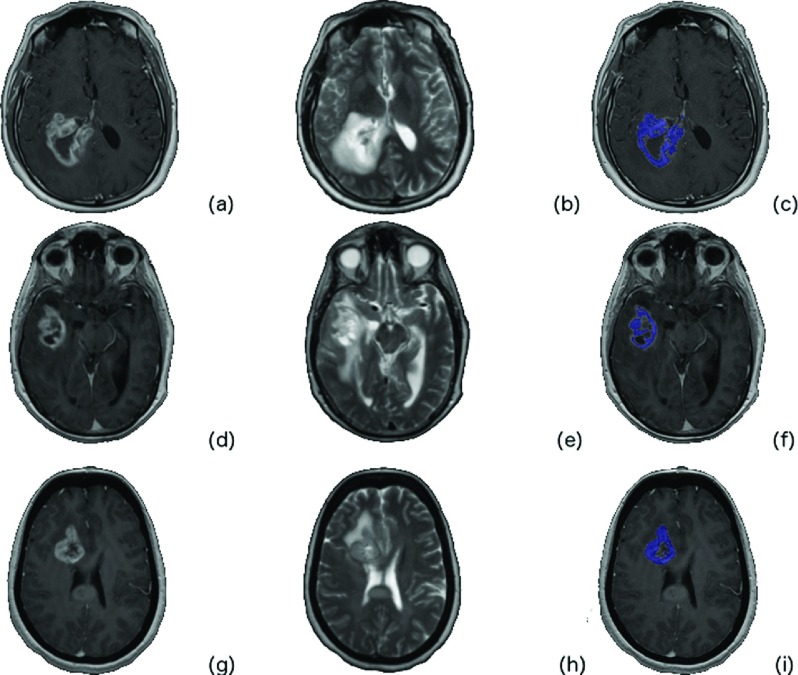
Glioblastoma cases with methylated MGMT GBM tumor. The *T*1 postcontrast [(a), (d), and (g)], the *T*2 [(b), (e), and (h)], and the tumor ROI overlaid (blue) on the *T*1 postcontrast are depicted [(c), (f), and (i)].

**FIG. 3.**
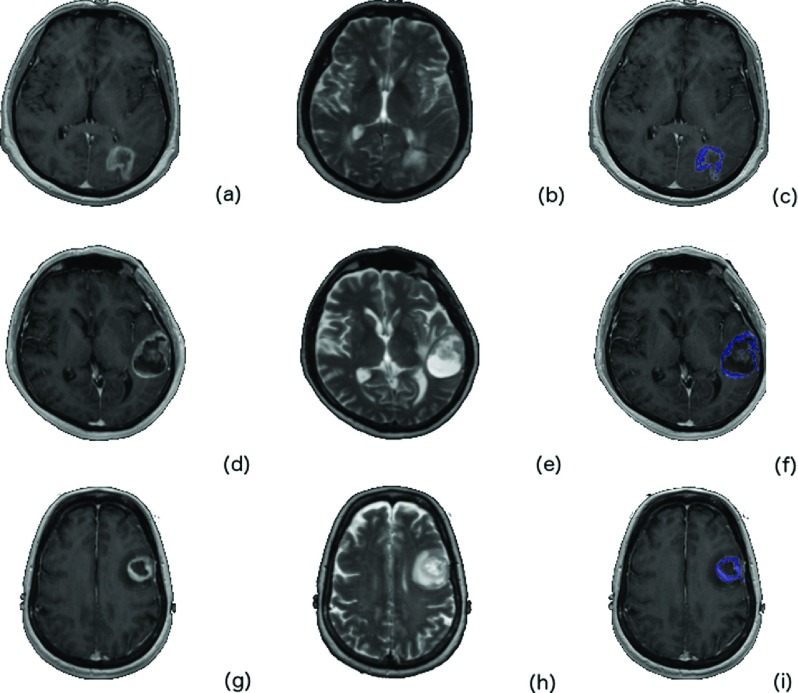
Glioblastoma cases with unmethylated MGMT GBM tumor. The *T*1 postcontrast [(a), (d), and (g)], the *T*2 [(b), (e), and (h)], and the tumor ROI overlaid (blue) on the *T*1 postcontrast are depicted [(c), (f), and (i)].

Figure 2 depicts a contrast-enhanced image of three different (by row) patients with MGMT methylated GBM and the corresponding ROI considered in this study. Figure 3 is from three different (by row) patients with MGMT unmethylated GBM.

#### 2.B.3. Intensity normalization

Even when using the same protocol and the same subject in the same scanner, MRI intensities can vary. Intensity variations are greater when different scanners are used. To minimize variability in image intensity, the intensities were normalized to the mean value of normal-appearing white matter utilizing the ICBM MR-*T*1 (International Consortium for Brain Mapping, template: 181 × 217 × 181 mm) atlas registered to the patient data^15^ utilizing the ANTs diffeomorphic registration.^16^ Subsequently the transformation was applied to the probabilistic WM map. All the pixels with probability of 0.9 belonging to WM were considered. Pixels corresponding to the bounding box encapsulating the tumor area expanded by ten pixels on each side were zeroed out. Subsequently, a linear transformation was implemented so the median value of WM was 1000.

#### 2.B.4. Texture features

Run length matrix (RLM)-based features capture the variability of intensity in a specified direction. A run is defined as a string of consecutive voxels that have the same gray level (GL) intensity in the specified direction. For each run, ten features were calculated.^17^ The mean of each feature over the four run length matrices (corresponding to four directions) was calculated, comprising a total of ten run length-based features (Long Run High Gray Level Emphasis, Long Run Low Gray Level Emphasis, Short Run High Gray Level Emphasis, Gray Level Nonuniformity, High Gray Level Run Emphasis, Long Run Emphasis, Low Gray Level Run Emphasis, Run Length Nonuniformity, Short Run Emphasis, and Short Run Low Gray Level Emphasis). The texture features were calculated based on an Insight Segmentation and Registration Toolkit (ITK)^18^ implementation.^19^

Gray level co-occurrence matrix (GLCM) is a well-established tool for characterizing the second order statistics of the spatial distribution of gray levels in an image.^20^ For each image, four GLCMs (corresponding to 4 directions) were calculated, and eight features (Cluster Prominence, Cluster Shade, Correlation, Energy, Entropy, Haralick Correlation, Inertia, Inverse Difference Moment) were derived, again using the ITK tool.^18^ The mean of each feature over the four GLCM directions was calculated.

Eighteen texture-filtered versions of the images were created after application of the texture filters to the initial images and averaging over the four directions for all the combinations of intensity binning (16, 32, 64, and 128) and window filter sizes (3 × 3, 5 × 5, 7 × 7, and 9 × 9) considered in this study.

The window filter size refers to sliding widow size used in order to create the texture-filtered versions of the images. For each window size, the co-occurrence and run length matrices were created, and subsequently the corresponding features were calculated. For each sliding window, the center pixel is replaced with these calculated feature values.

The features were calculated on a 2D basis. For each of the tumor ROIs, the mean, median, standard deviation, and the 10th and 80th percentiles were calculated for the filtered version of the image over the volume of the tumor. The intensity of the images was also evaluated as a feature. The volume of the enhancing region after the manual correction was also included as a candidate imaging biomarker.

#### 2.B.5. Classification scheme

Feature selection was applied to identify useful texture features and eliminate redundant ones using the Ridge regression technique,^21^ which is an *L*2 regularization-based technique that biases a model toward lower complexity (less coefficients), with the goal of preventing overfitting and improving generalization.

Two supervised machine-learning classifiers were tested: support vector machine (SVM)^22^ and the RFC.^23^ The SVM classifier is based on transformation of feature space to a higher dimension space where a separating hyperplane maximizes the distance between classes; they are known for good generalization. In this study, we used a Gaussian radial basis function kernel, and we tested a range of values for *C* and *σ* (larger *C* values mean that a higher penalty is assigned to the misclassified cases, and *σ* defines the impact of a single training example).

An RFC is an ensemble of decision trees. Each tree is typically trained with random subsets of the training set and features (known as bootstrap aggregation or “bagging”) to improve classifier generalization. Training aims to identify the set of tests in each decision tree that separates the data into different classes. A test example traverses different trees by applying the tests according to the path from the root node to the leaf it traverses. When a terminal (“leaf”) node is reached, the tree votes for the class assigned to this node in the training stage. The final decision for a test example is obtained by selecting the class with the number of votes exceeding a threshold, typically the majority of votes. In total, four supervised classification schemes were studied, *T*2-based texture combined with SVM (referred to as *T*2-SVM), *T*1-based texture combined with SVM (referred to as *T*1-SVM), *T*2-based texture combined with RFC (referred to as *T*2-RFC), and *T*1-based texture combined with RFC (referred to as *T*1-RFC). Finally the features selected to the best-performing classification schemes were combined in order to test the performance of the classification schemes when *T*1- and *T*2-based texture features are utilized (referred to as *T*1/2-SVM and *T*1/2-RFC).

#### 2.B.6. Parameter selection

The selection of the ROI filter window size and number of GL bins was based on the area under ROC curve (*A_z_*) using a stratified fivefold crossvalidation method for all the classification schemes in this study. Grid search was performed in order to identify the set of optimal parameters on the basis of highest *A_z_*. In the case of the SVM classifier, the parameters *C* and *σ* were also determined via grid search, while the same applies in case of the RFC for the number of decision leafs. In this study, the grid consisted of 4 different filter sizes (3, 5, 7, and 9) and 4 different GL bins (16, 32, 64, and 128). For SVM, the values of *C* tested were 10^−4^, 10^−3^, …, 10^3^, 10^4^, and for *σ*, 10^−3^, …, 10^2^ in log space and, in the case of RFC, we tested 1 through 100 trees in steps of 1.

#### 2.B.7. Statistical analysis

The *A_z_* was calculated to assess performance of the different classification schemes^24^ using a stratified fivefold crossvalidation method. *A_z_* is an effective method of evaluating the performance of diagnostic test that combines the measures of sensitivity and specificity. The pROC (ver. 1.7.3) package^25^ for *R* ver. 3.2.1 was used for the receiver-operator characteristic curve and *A_z_* calculations, with the DeLong method^26^ used for statistical comparison of ROCs.

## 3. RESULTS

Tables I–IV capture all the classification schemes (supervised classifier and selected features) with area under ROC greater or equal to 0.75 when applied on a dataset comprised of 155 patients (66 methylated and 89 unmethylated tumors). *A_z_* values closer to 1 indicate better overall diagnostic performance of scheme evaluated.

The number of selected features is reported as well as the parameters used in each of the supervised classification schemes (i.e., the number of estimators for the RFC and the parameters *C* and *σ* for SVM). The size and the gray level utilized to calculate the texture features are also reported. The *A_z_* observed was 0.85 (95% CI: 0.78–0.91) for texture features originating from the *T*2 images when utilizing the SVM classifier, yielding at the optimal threshold of the ROC curve, sensitivity of 0.803 and specificity of 0.813. Four features, all from co-occurrence based texture features, were selected. The second best AUC was observed again for texture features originating from the *T*2 image for the RFC. In the case of SVM combined with the texture features extracted from the *T*1 postcontrast images, the AUC was less than 0.76 (Table IV). The number of selected features varied from 2 to 13.

The DeLong method^26^ was used to statistically compare the ROC curves obtained from each of the classification schemes. The analysis found statistically significant differences between *T*2 texture classification by SVM (*T*2-SVM) versus *T*1-RFC and *T*1-SVM (*p* <0.001), but no difference when compared to *T*2-RFC.

Table V summarizes the selected features for the best-performing classification schemes. The selected features originating from both the *T*1- and *T*2-based texture features (Table VI) were also combined and evaluated with the SVM and RFC classifiers. The area under ROC for *T*1/2-SVM was 0.812 (95% CI: 0.632–0.951), while *T*1/2-RFC was 0.78 (95% CI: 0.580–0.948).

In order to attempt a connection between the important variables to biologic mechanisms, a description of the physical meaning of each texture feature is given in Table VI.

**FIG. 4.**
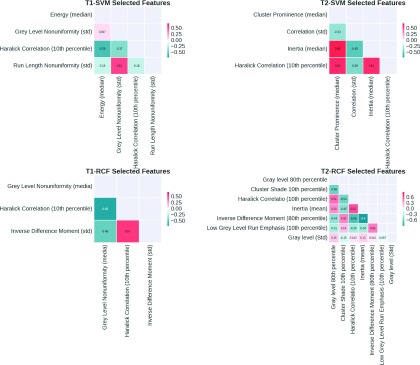
Correlations of the selected features for the best-performing classification schemes.

Figure 4 captures the correlation of the selected features for the best-performing classification schemes.

**FIG. 5.**
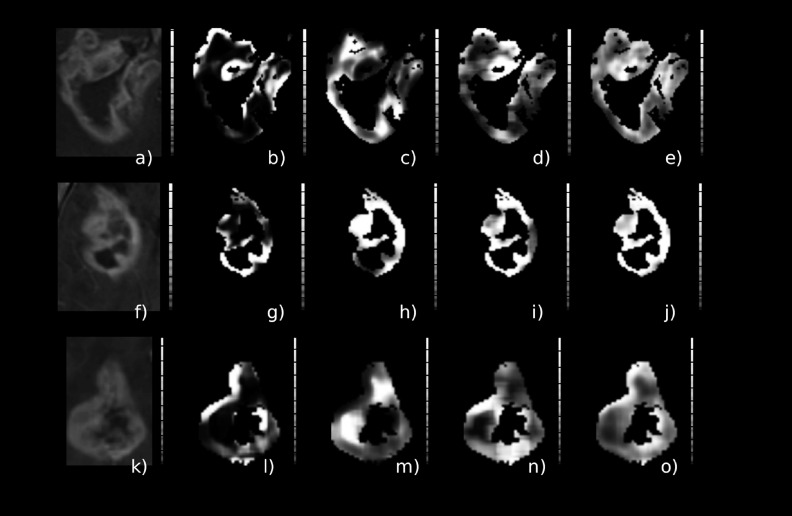
Glioblastoma cases with methylated MGMT GBM tumor. Texture features corresponding to the best-performing classification scheme (*A_z_* = 0.850), cluster prominence [(b), (g), and (l)], correlation [(c), (h), and (m)], Haralick correlation [(d), (i), and (n)], inertia [(e), (j), and (o)], and corresponding tumor ROI [(a), (f), and (k)].

**FIG. 6.**
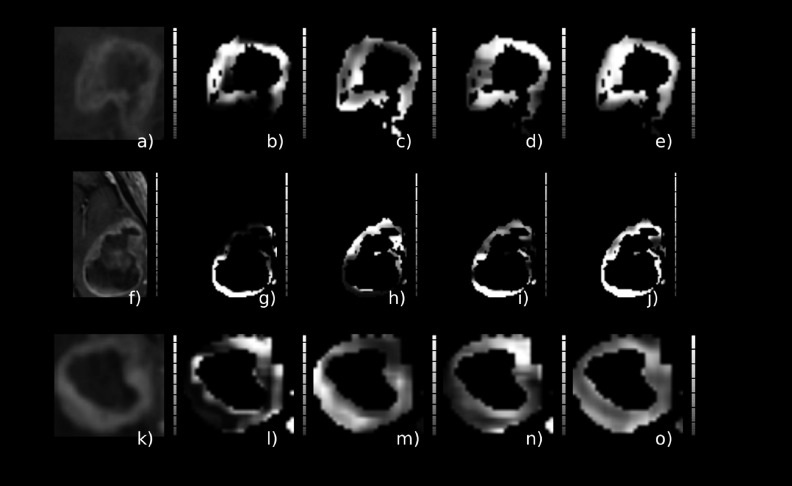
Glioblastoma cases with unmethylated MGMT GBM tumor. Texture features corresponding to the best-performing classification scheme (*A_z_* = 0.850), cluster prominence [(b), (g), and (l)], correlation [(c), (h), and (m)], Haralick correlation [(d), (i), and (n)], inertia [(e), (j), and (o)], and corresponding tumor ROI [(a), (f), and (k)].

Figures 5 and 6 depict the texture-filtered images corresponding to the selected features for the best-performing classification scheme for an MGMT unmethylated and MGMT methylated case, respectively.

## 4. DISCUSSION

This paper presents a scheme for differentiating between MGMT methylated and unmethylated GBMs based on MRI image textures. MGMT methylation is important for predicting the treatment response to chemotherapy with an alkylating agent. Imaging biomarkers based on intensity, and first- and second-order statistics are utilized for this purpose. Machine learning and second-order texture features have been used previously in many MRI-based studies.^28^ In this study, two different categories of texture features (co-occurrence and run length based) were utilized as well as first-order statistics extracted from the tumor regions of *T*1 postcontrast and *T*2 images. The volume of the enhancing tumor region was also evaluated as an imaging biomarker.

The aim of this study is to identify features that can classify regions of the tumor that are methylated or unmethylated. Once these features are identified, they can lead to parametric maps that can highlight methylated or unmethylated tumor regions. We anticipate that this can impact treatment efficacy, since knowing if recurrent tumor is methylated or not is important, as repeated biopsy is not a preferred action.

The SVM-based classification of texture patterns is a very promising approach for providing a prediction of the MGMT methylation status of the brain tumors. An *A_z_* of 0.850 (95% CI: 0.782–0.913) was observed when combining the SVM classifier with co-occurrence based texture features (Table III), yielding at the optimal threshold of the ROC curve, sensitivity of 0.803 and specificity of 0.813. Among the features selected for the classification schemes, the Haralick correlation feature was selected in all four best-performing schemes for both the *T*1 and *T*2 images.

When supervised classifiers were used with both *T*1- and *T*2-weighted images, performance was poorer than the best schemes corresponding to *T*2-SVM and *T*2-RFC. Thus, a feature originating from *T*1 images combined with *T*2-based feature seems to be reducing performance of the supervised classification schemes especially in the case of the RFC.

Since the nature of the underlying texture difference between the radiologic expressions of methylated and unmethylated tumors is unknown, we first compared the features for each individual direction. Since no significant difference was found based on direction, we averaged them together, which allowed us to reduce the number of features.

Based on the selected features, and their physical meaning, it seems that the features that capture the uniformity as well as the symmetry in the ROI were selected for all the best models considered in this study. Thus it seems that there is a difference in uniformity between methylated and unmethylated tumors that the combination of these features is able to capture.

MGMT unmethylated tumors have been shown to have larger volumes for both enhancing (*T*1 postcontrast) and *T*2/flair hyperintensity.^8^ The enhancing volume was one of the imaging biomarkers considered in the study, but volume was not found to be useful as a predictor of methylation status in any of the best-performing schemes.

Generally, the classification accuracy of our proposed method is comparable with other reported approaches investigating imaging biomarkers extracted from MRI datasets. Ahn *et al.*^11^ reported *A_z_* value of 0.756 utilizing *Ktrans* as imaging biomarker (cutoff = 0.086 mm^−1^). Recently ADC was also highlighted as a potential surrogate biomarker for MGMT status detection,^10,29^ though Thiele *et al.*^7^ demonstrated strong dependency on preprocessing technique. Furthermore, the majority of previous studies had relatively small datasets (less than 50 patients) and evaluated individual characteristics without investigating potential combinations. By combining imaging biomarkers that describe different aspects of tumor appearance, we can build a more accurate model to predict MGMT methylation. Inclusion of other MR imaging approaches, such as ADC or dynamic susceptibility contrast, could further improve the accuracy of the proposed technique.

We also studied a number of visual/qualitative measures [enhancing tumor margin (well or poorly defined); enhancement pattern (ring, nodular, or mixed enhancement); presence of edema, cystic regions, necrosis, and nonenhancing tumor; and heterogeneity of the signal intensity on the *T*2-weighted images], but none was correlated with MGMT methylation status, which is in agreement with previous results.^11,12^ In particular, no association was found between ring enhancement and methylation status with almost half of the cases from both categories presenting the ring enhancement pattern.^8,27^ Classification schemes, such as we have shown here, may also be applied to predict other molecular biomarkers such as isocitrate dehydrogenase mutation or 1p19q status in both high-grade and low-grade tumors. A recent publication indicated that when gliomas were classified into five principal groups on the basis of three tumor biomarkers, the resulting groups had different ages at onset, overall survival, and associations with germline variants, implying distinct mechanisms of pathogenesis where imaging-based biomarkers can play a crucial role.^30^

Regarding clinical implementation, there are two main challenges for the translation of the proposed approach to the clinical practice. First, the semiautomated step requires feature extraction, which requires a fair amount of computational power and someone with expertise in implementing the processing. Second, the time required for the registration between the atlas and the images is rather long (in total, ∼45 min of processing time for each patient was required).

We recognize that different acquisition protocols could affect the proposed scheme since different image resolution can affect the texture extraction process. Theoretically, increasing the magnetic field strength from 1.5 to 3 T roughly doubles the signal-to-noise ratio, thus providing higher contrast to noise leading to better differentiation of gray/white matter and other tissues. On the other hand, 3 T MRI scanners have been reported to have artifacts that can affect contrast.^31^ Artifacts can also affect texture appearance. Thus, phantom studies should be performed in order to fully understand the effect of scanner field strength on texture appearance.

Texture features derived from the co-occurrence matrix have been proven to enable discrimination of different patterns close to the resolution limits for the smallest structures of physical texture even for datasets that are heterogeneous with regard to different acquisition parameters, including spatial resolution.^32^ Larger datasets are needed in order to investigate the generalizability of the proposed scheme and test the robustness against different dataset origins. We believe that inclusion of larger datasets will lead to improved performance.

## 5. CONCLUSION

Supervised machine learning schemes based on SVM and texture features from MR images can be used to predict MGMT methylation status in preoperative GBM tumors, providing a noninvasive imaging correlate for this important biomarker. In contrast, visual inspection of a number of traditional MRI parameters does not differentiate MGMT methylation status. These results suggest that this approach should be pursued further—particularly in a larger, more heterogeneous cohort of patients in order to validate these findings.

## Figures and Tables

**TABLE I.** Results from RFC with feature extracted from *T*1 postcontrast images (best-performing system is in bold).

Number of selected features	Classifier parameter (estimator)	Window filter size	GL	*A_z_*
2	50	3	16	0.719
				95% CI: 0.551–0.742
**3**	**10**	**3**	**64**	**0.756**
				95% CI: 0.613–0.794
5	100	5	32	0.706
				95% CI: 0.551–0.747

**TABLE II.** Results from RFC with feature extracted from *T*2 images (best-performing system is in bold).

Number of selected features	Classifier parameter (estimator)	Window filter size	GL	*A_z_*
10	50	3	16	0.820
				95% CI: 0.662–0.849
7	10	3	64	0.700
				95% CI: 0.521–0.732
13	10	3	128	0.756
				95% CI: 0.432–0.798
**7**	**100**	**5**	**32**	**0.840**
				95% CI: 0.757–0.892

**TABLE III.** Results from SVM with feature extracted from *T*2 images (best-performing system is in bold).

Number of selected features	Classifier parameter (estimator)	Window filter size	GL	*A_z_*
4	*C*: 10.0,	3	16	0.830
	*σ*: 0.1			95% CI: 0.637–0.867
**4**	***C*: 10.0,**	**3**	**32**	**0.850**
	*σ***: 0.01**			95% CI: 0.782–0.913
4	*C*: 1.0,	3	64	0.780
	*σ*: 1 × 10^−03^			95% CI: 0.594–0.804
4	*C*: 1.0,	5	16	0.780
	*σ*: 1.1 × 10^−03^			95% CI: 0.633–0.821
4	*C*: 1.0,	5	16	0.800
	*σ*: 1.0			95% CI: 0.512–0.822
8	*C*: 10.0,	5	16	0.760
	*σ*: 1 × 10^−04^			95% CI: 0.422–0.824
4	*C*: 100.0,	5	64	0.750
	*σ*: 1 × 10^−02^			95% CI: 0.410–0.816

**TABLE IV.** Results from SVM with feature extracted from *T*1 postcontrast images (best-performing system is in bold).

Number of selected features	Classifier parameter (estimator)	Window filter size	GL	*A_z_*
3	*C*: 1.0,	3	16	0.760
	*σ*: 1 × 10^−04^			95% CI: 0.475–0.813
**4**	***C*: 100.0,**	**3**	**32**	**0.763**
	*σ***: 1 × 10**^**−04**^			95% CI: 0.383–0.826
4	*C*: 1.0,	3	64	0.747
	*σ*: 1 × 10^−04^			95% CI: 0.482–0.821

**TABLE V.** Selected features for the best-performing classification systems (best-performing system is in bold). The individual feature *A_z_* is also provided.

Supervised scheme	Selected features
*T*1-SVM	Energy (median, *A_z_* = 0.586), gray level nonuniformity (std, *A_z_* = 0.690), Haralick correlation (tenth percentile, *A_z_* = 0.690), run length nonuniformity (std, *A_z_* = 0.605)
*T*1-RFC	Gray level nonuniformity (median, *A_z_* = 0.586), Haralick correlation (tenth percentile, *A_z_* = 0.586), inverse difference moment (std, *A_z_* = 0.586)
***T*2-SVM**	**Cluster prominence (median, *A*_z_** = **0.677), correlation (std, *A*_z_** = **0.623), inertia (median, *A*_z_** = **0.648), Haralick correlation (tenth percentile, *A*_z_** = **0.575)**
*T*2-RFC	Gray level (80th percentile, ***A*_z_** = **0.635**), cluster shade (tenth percentile, ***A*_z_** = **0.679**), Haralick correlation (tenth percentile, ***A*_z_** = **0.575**), inertia (mean, ***A*_z_** = **0.686**), inverse difference moment (80th percentile, ***A*_z_** = **0.642**), long run low gray level emphasis (tenth percentile, ***A*_z_** = **0.757**), gray level (std, ***A*_z_** = **0.728)**

**TABLE VI.** Physical meaning of the selected co-occurrence and run length features.

Feature	Physical meaning
Energy	Measure of uniformity
Gray level nonuniformity	Measures the similarity of gray level values throughout the image. Low values of this measure mean that the gray level values are similar throughout the ROI
Run length nonuniformity	Measures the similarity of the length of runs through out the image. The run length nonuniformity is expected to be small if the run lengths are alike through out the image
Cluster prominence	Cluster prominence is a measure of asymmetry. High values mean that the image is less symmetric. Furthermore, low values mean that there is an increased concentration in the co-occurrence matrix around the mean. For an MRI image, a low cluster prominence value indicates small variation in gray level
Haralick correlation	Correlation is a measure of gray level linear dependence between the pixels at the specified positions relative to each other
Cluster shade	Cluster shade is a measure of the skewness of the matrix and is believed to gauge the perceptual concepts of uniformity. When the cluster shade is high, the image is asymmetric
Inertia	The inertia texture feature is very sensitive to large differences occurring inside the co-occurrence matrix. In terms of gray level intensity, this means that highly contrasted regions will have a high inertia and homogeneous regions will have a low inertia
Inverse difference moment	Inverse difference moment is influenced by the homogeneity of the image. The result is a low inverse difference moment value for inhomogeneous images and higher value for homogeneous images
Long run low gray level emphasis	Measures the joint distribution of long runs and high gray level values. The long run low gray level emphasis is expected to be large for images with many long runs and high gray level values

Note: None of the visual measurement performed was able to predict MGMT methylation status (Ref. 27).
